# Dorsal root ganglia neurite outgrowth measured as a function of changes in microelectrode array resistance

**DOI:** 10.1371/journal.pone.0175550

**Published:** 2017-04-13

**Authors:** Jordan M. Renna, Jessica M. Stukel, Rebecca Kuntz Willits, Erik D. Engeberg

**Affiliations:** 1 Department of Biology, University of Akron, Akron, Ohio, United States of America; 2 Department of Biomedical Engineering, University of Akron, Akron, Ohio, United States of America; 3 Ocean & Mechanical Engineering Department, Florida Atlantic University, Boca Raton, Florida, United States of America; University of Palermo, ITALY

## Abstract

Current research in prosthetic device design aims to mimic natural movements using a feedback system that connects to the patient's own nerves to control the device. The first step in using neurons to control motion is to make and maintain contact between neurons and the feedback sensors. Therefore, the goal of this project was to determine if changes in electrode resistance could be detected when a neuron extended a neurite to contact a sensor. Dorsal root ganglia (DRG) were harvested from chick embryos and cultured on a collagen-coated carbon nanotube microelectrode array for two days. The DRG were seeded along one side of the array so the processes extended across the array, contacting about half of the electrodes. Electrode resistance was measured both prior to culture and after the two day culture period. Phase contrast images of the microelectrode array were taken after two days to visually determine which electrodes were in contact with one or more DRG neurite or tissue. Electrodes in contact with DRG neurites had an average change in resistance of 0.15 MΩ compared with the electrodes without DRG neurites. Using this method, we determined that resistance values can be used as a criterion for identifying electrodes in contact with a DRG neurite. These data are the foundation for future development of an autonomous feedback resistance measurement system to continuously monitor DRG neurite outgrowth at specific spatial locations.

## Introduction

The field of neuroprosthetics has the potential to substantially improve the lives of millions of people worldwide [1, 2]. However, the development of neuroprostheses has been relatively slow because of the time and costs associated with device approvals. To reduce costs in development and provide solutions that will be more likely to succeed in clinical trials, preclinical studies require the development of noninvasive research tools to gather meaningful data pertaining to nerve function and regeneration in the presence of realistic microenvironments [3]. One method of interfacing with neurons to gather signals temporally and noninvasively is through the use of microelectrode arrays. Microelectrode arrays have been utilized with varied success to deliver or measure electrical signals both *in vitro* and *in vivo* from neurons [4].

Microelectrode arrays usually consist of 3–512 extracellular electrodes with an even spatial distribution where each electrode typically samples the voltage in its microenvironment. One neuron can generate voltage deflections within the extracellular environment that can be detected more than 300 micrometers away from the neuron *in vivo* [5]. These signals can be detected on every electrode and as the signal dissipates radially through the environment the amplitude decreases. In complex neural networks, each extracellular electrode can sample signals from a large number of neurons that vary in distance from the electrode; for review see [6]. Because each electrode samples multiple neural signals within one voltage measurement, accurately interpreting the data in real-time is extremely difficult or nearly impossible. This setup creates numerous challenges for using voltage measurements to monitor the stability of the neural-electrode interface in a dynamic environment over long periods of time. In contrast, the impedance of each electrode has more predictable changes temporally. For example, cortical neurons and fibroblasts cultured on a 3D matrix with a microarray have been shown to increase contact with microelectrodes over a period of 8 days, increasing electrode impedance [7]. Electrode arrays have been used for over 40 years to gain insight into neuronal signaling through delivery or measurement of electrical information, usually in the form of voltage [8]. A critical feature of an electrode is its impedance, or frequency-dependent resistance. When tissue contacts electrodes, the interaction with cells and proteins typically increases the electrode resistance. Consequently, electrophysiologists have used impedance and resistance readings to assess the cleanliness, quality and viability of electrode arrays between experiments. However, these changes in electrical characteristics can also be exploited to determine how cells are interfacing with the microelectrode arrays during experiments [7]. Due to the simplicity of measuring resistance in comparison to impedance we propose using extracellular electrode resistance as a tool for assessing a stable nerve-electrode interface, where any significant changes in electrode resistance between the ground electrode and the experimental electrode would be directly correlated to changes in the nerve-electrode interface at a specific spatial location.

Sensory neurons of the peripheral nervous system are in close proximity to the spinal cord and are a practical target for neuroprosthetic implants [9, 10]. Previous research explored the possibility of measuring electrode impedance *in vivo* on floating microelectrode arrays in cat ventral roots, which contain the axons of motor neurons [11]. However, consistent results were difficult to achieve due to challenges in maintaining tissue contact with the microelectrode array. Here we demonstrate that consistent resistance measurements can be obtained from DRG neurons seeded on a microelectrode array and determined a criterion sufficient to confirm DRG contact with an electrode with 100% accuracy after two days of culture.

## Materials and methods

### DRG culture and preparation

Embryonic day-nine-chick DRG were utilized throughout the experiments. The Public Health Service Policy on Humane Care and Use of Laboratory Animals does not require IACUC approval for use of chick embryos prior to embryonic day 19. However, all embryos were treated humanely. Chick DRG were extracted from specific pathogen free (SPF) eggs (Sunrise Farms, Catskill, NY). Excess tissue was cleaned from the DRG in calcium-containing Hank’s balanced salt solution (all cultured reagents obtained from Sigma, St Louis, unless otherwise stated). The 60PedotMEA200/30iR-Au-gr glass microelectrode array (MEA; Multichannel Systems, Germany) was first autoclaved for 20 min and then coated with 0.1 mg/mL rat-tail extracted type I collagen [12, 13] for 30 min and rinsed with phosphate buffered saline (MP Biomedicals, Solon, OH). Approximately 0.2 mL complete media (F12K) supplemented with 20% fetal bovine serum and 50 ng/mL nerve growth factor (BD, Columbus, NE) was added to the MEA chamber. DRG were seeded onto the MEA, with a gas permeable barrier placed over the top. DRG-MEAs were placed in an incubation chamber for two days at 37°C with 5% C0_2_.

### Microelectrode array and resistance measurements

Experiments were designed to assess the ability of changes in electrode resistance values to accurately detect when a DRG neurite contacts an electrode. Each MEA was comprised of 59 carbon nanotube extracellular electrodes 30 μm in diameter and spaced out center to center 200 μm apart in an 8 x 8 grid. Contact pads and tracks were gold plated, and only the carbon nanotube electrodes protruded from the glass. MEAs were placed into an MEA-60 amplifier system (Multichannel Systems) and a Fluke 87V Industrial Multimeter (accuracy of ± 0.2% MΩ; Fluke Corporation, Everett, WA) was used to measure resistance values. One lead was connected to the MEA-60 amplifier ground pin and the other lead was connected to the pin of each electrode one at a time. Resistance measurements were made three times from each electrode, and the average resistance value was used. Resistance measurements were made with culture media prior to DRG seeding and then again after the two day incubation period. High resolution phase contrast photomicrographs were obtained to visually identify electrodes in contact with DRG processes with a Zeiss Observer Z1 microscope. Eight individual experiments were conducted and in two cases the DRG soma did not adhere to the MEA (n = 6). For statistical significance analysis a one-way ANOVA was used to evaluate each individual experiment comparing the resistance by group (SOMA, DRG neurite, CLEAN, UNCLEAR) with Tukey post hoc test. A p value less than 0.05 was considered significant. Standard Error is reported. The aggregate result for each experiment (n = 6) was also compared between each group using one-way ANOVA with Tukey post hoc test; p<0.05 was considered significant.

## Results

**Fig 1:**
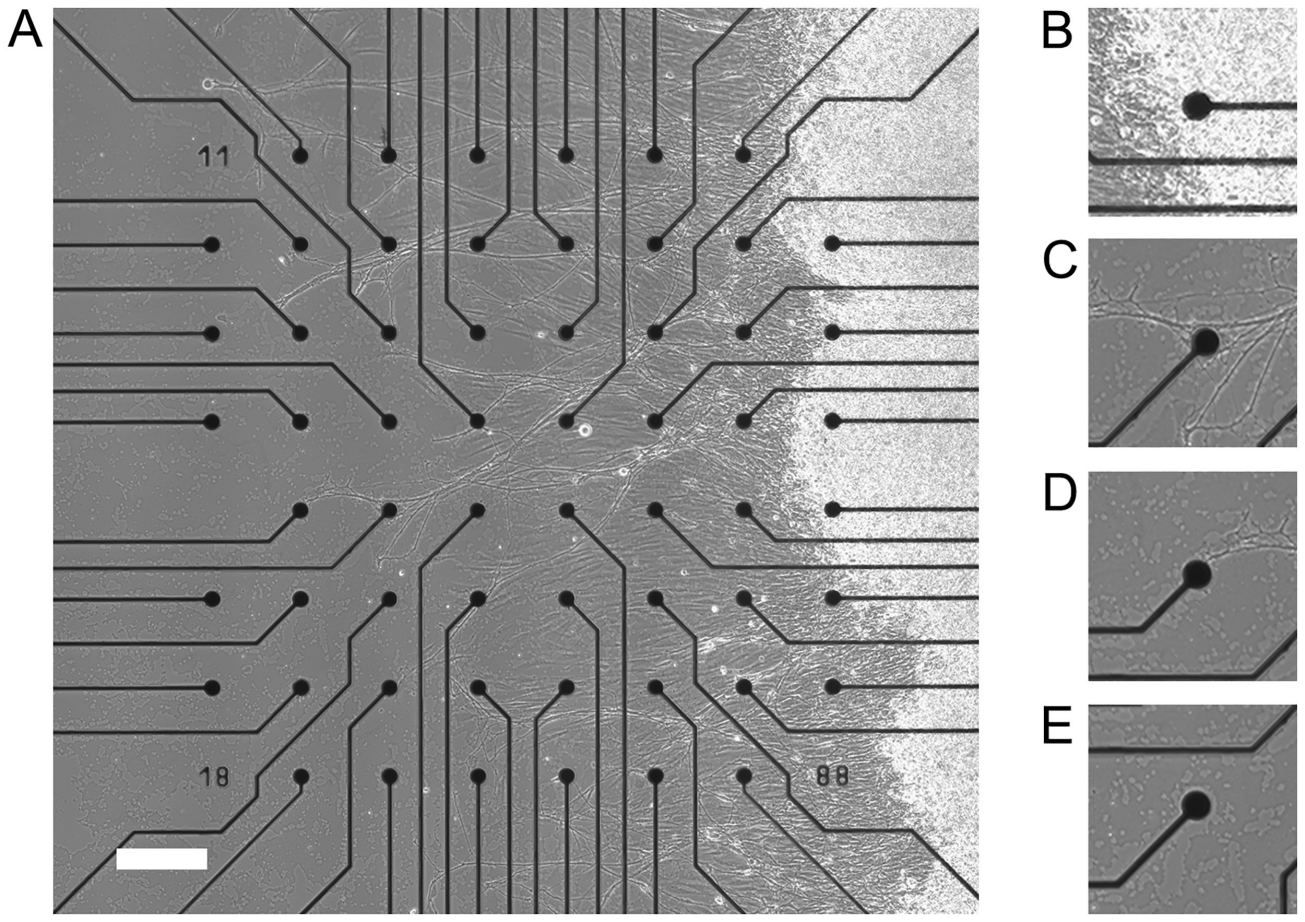
High resolution phase contrast photomicrograph of DRG tissue seeded on a microelectrode array after a 2 day culture. (A). 59 extracellular electrodes were visually classified as having contact with a DRG soma (B) or a DRG neurite (C). For some electrodes it was unclear if there was DRG neurite contact (D) and electrodes devoid of contact with DRG tissue were classified as CLEAN (E). The majority of electrodes classified as CLEAN were located on the opposite side of the array from where the DRG tissue was located (left side of A; scale bar = 200 μm).

Resistance values were measured from each electrode prior to DRG plating and culturing. After a period of 2 days, resistance values were measured for each electrode and the change in resistance was calculated. Additionally, each electrode on the 60 channel microelectrode array was imaged (Fig 1A). Using a photomicrograph, for each electrode on the MEA, it was determined if a SOMA was in contact with the electrode, if a DRG neurite was in contact with the electrode, if it was UNCLEAR if there was anything in contact with the electrode, or if the electrode was CLEAN (Fig 1B–1E). Electrode resistance values were then grouped by classification (SOMA, DRG neurite, CLEAN, or UNCLEAR), and we compared the average change in resistance between each of these groups. Electrodes classified as contacted by a SOMA were always nearest to where the DRG was seeded, and CLEAN electrodes were almost always the electrodes furthest away from the DRG soma.

**Fig 2:**
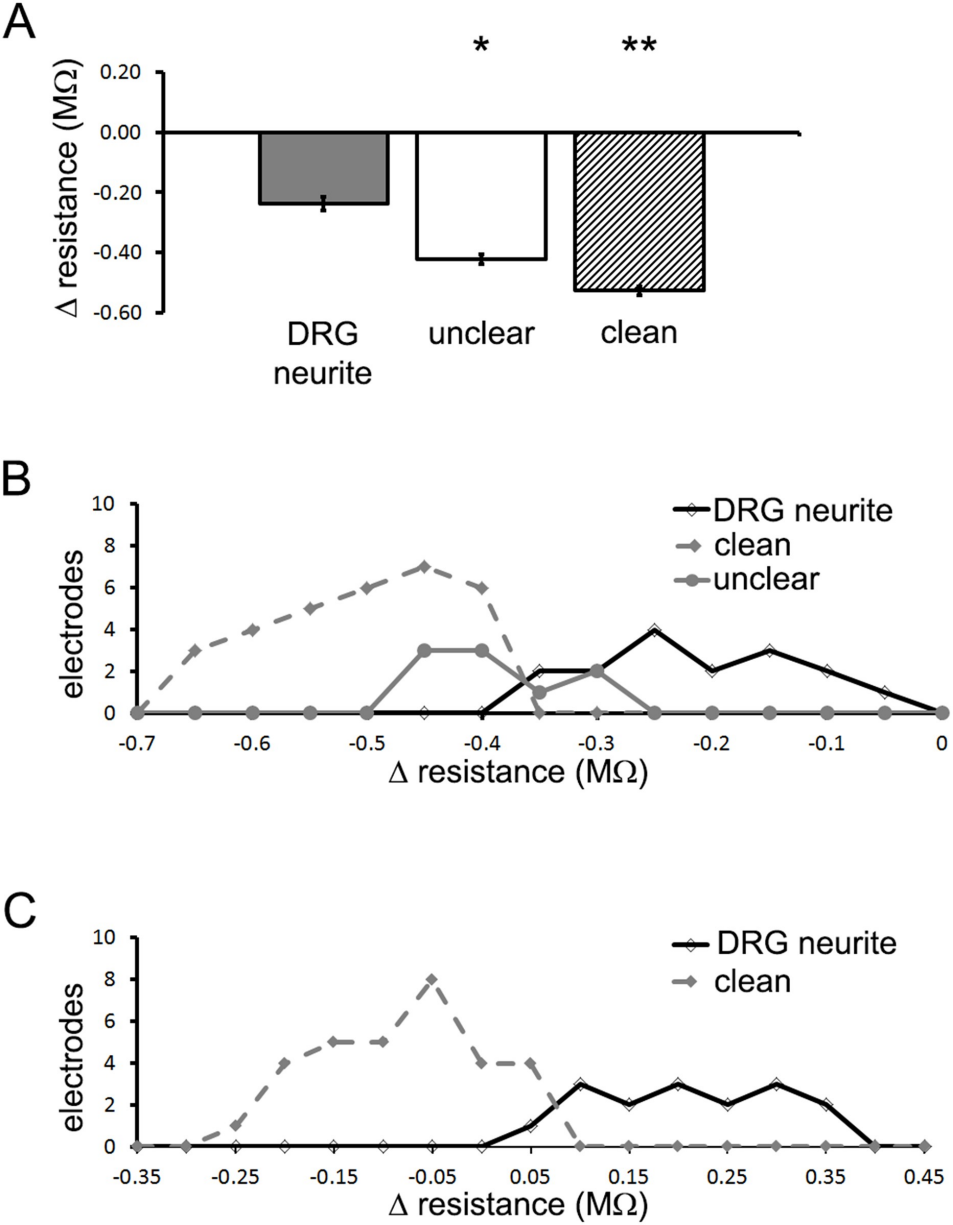
Electrode resistance values can reflect DRG neurite contact with electrode. In a single experiment resistance values from electrodes were averaged by classification (DRG neurite, UNCLEAR, or CLEAN). The soma in this experiment was not placed in contact with any electrodes. (A) The change in resistance for electrodes in contact with a DRG neurite (-0.24 ± 0.02 MΩ; n = 16; p < 0.001; F = 69.685) were significantly less than for electrodes classified as UNCLEAR (-0.42 ± 0.02 MΩ; n = 19; p < 0.001) or CLEAN (-0.53 ± 0.01 MΩ; n = 31 electrodes). (B) The distribution of the change in electrode resistances for CLEAN electrodes, UNCLEAR electrodes and electrodes contacted by a DRG neurite were plotted in a frequency curve (0.5 MΩ bins). (C) The average change in resistance of UNCLEAR electrodes was background subtracted across all electrodes in a single experiment. This did not change the relationship between resistance values of electrodes contacted by DRG neurites and CLEAN electrodes. However it allowed for a comparison of resistance values across experiments.

In an experiment run over a 2 day period of time, resistance values on CLEAN electrodes without a DRG neurite or a DRG soma in contact with an electrode decreased (Fig 2A; -0.53 ± 0.01 MΩ; n = 31 electrodes). Resistance values on electrodes that were contacted by a DRG neurite only decreased by about half (-0.24 ± 0.02 MΩ; n = 16; p < 0.001; F = 69.685) and when it was not clear if there was a DRG neurite in contact with the electrode (UNCLEAR) resistance values decreased, but not as significantly as when an electrode was CLEAN (-0.42 ± 0.02 MΩ; n = 9; p < 0.001). For a single experiment, the distribution of electrode resistances for CLEAN electrodes, UNCLEAR electrodes, and electrodes contacted by a DRG neurite were plotted in a frequency curve (Fig 2B). Any electrode that was CLEAN had a decrease in resistance of at least -0.35 MΩ.

**Fig 3:**
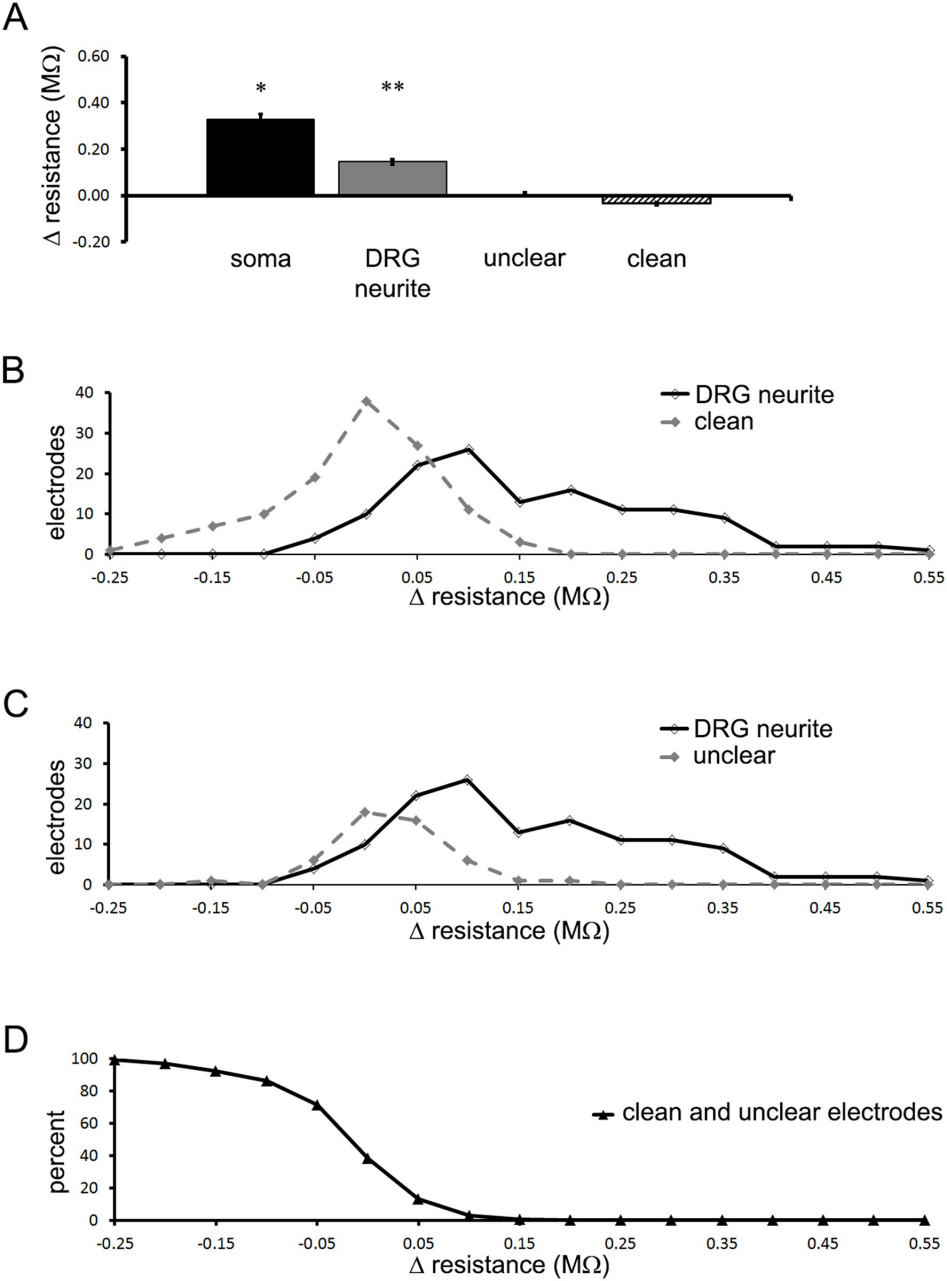
A criterion of 0.15 MΩ relative to CLEAN electrodes would predict with 100% accuracy electrodes contacted by a DRG process. (A) Resistance values background subtracted to UNCLEAR electrodes within experiments were averaged across experiments. The change in resistance for electrodes in contact with a DRG soma (0.34 ± 0.06 MΩ; p < 0.001; n = 45 electrodes; UNCLEAR n = 49 electrodes; experiment n = 6) and a DRG neurite (0.16 ± 0.04 MΩ; p < 0.05; n = 130 electrodes) were similar to electrodes classified as CLEAN (-0.03 ± 0.01 MΩ; p > 0.05; n = 120). (B) The distribution of the change in electrode resistances for CLEAN electrodes and electrodes contacted by a DRG neurite for all experiments were plotted in a frequency curve (0.5 MΩ bins). Only electrodes contacted by a DRG neurite had a change in resistance of 0.15 MΩ or more when compared with CLEAN electrodes. (C) The distribution of the change in electrode resistances for electrodes contacted by a DRG neurite and for electrodes in which it was UNCLEAR were plotted in a frequency curve (0.5 MΩ bins). (D) The percentage of electrodes not contacted by a DRG soma or a DRG neurite (CLEAN and UNCLEAR) plotted as a function of the change in resistance. A change of 0.15 MΩ would predict contact with DRG tissue with extremely high accuracy (nearly 100%).

The change in resistance over time on CLEAN electrodes and UNCLEAR electrodes varied from experiment to experiment (-0.12 ± 0.11 MΩ and -0.09 ± 0.11 MΩ respectively). For each experiment we then background subtracted the average change in resistance of the UNCLEAR electrodes with all of the other electrodes within that experiment, and compared the change in resistance across all experiments (see population data in Fig 3A). The relationship between the frequency distribution curves for the CLEAN electrodes and the electrodes contacted by a DRG neurite were similar to the raw data (Fig 2C).

Across all experiments the largest change in resistance relative to the UNCLEAR electrodes occurred when the DRG soma was seeded in contact with an electrode (data for each experiment in Table 1; averaged data in Fig 3A; 0.34 ± 0.06 MΩ; p < 0.001; DRG soma n = 45 electrodes; UNCLEAR n = 49 electrodes; experiment n = 6). Electrodes contacted by a DRG neurite changed resistance by 0.16 ± 0.04 MΩ relative to UNCLEAR electrodes (p < 0.05; n = 130 electrodes) and clean electrodes had a -0.03 ± 0.01 MΩ change in resistance relative to UNCLEAR electrodes (p > 0.05; n = 120). When comparing the CLEAN electrodes (Fig 3B) and UNCLEAR electrodes (Fig 3C) with the electrodes contacted by a DRG neurite, the largest change in resistance recorded was 0.13 MΩ. Plotting the percentage of electrodes not contacted by a DRG soma or a DRG neurite (CLEAN and UNCLEAR) as a function of the change in resistance (Fig 3D) illustrates that a criterion of 0.15 MΩ would accurately predict that an electrode had been contacted by a DRG neurite.

**Table 1:** The average change in resistance (MΩ) of each classified electrode after background subtraction for all six experiments (SEM in brackets).

	SOMA	Neurite	Clean	Unclear
**Experiment 1**	**0.45 (-0.04)**	**0.27 (0.03)**	**-0.01 (0.01)**	**0.00 (0.01)**
**Experiment 2**	**0.20 (0.02)**	**0.05 (0.01)**	**-0.02 (0.01)**	**0.00 (0.01)**
**Experiment 3**	**0.33 (0.03)**	**0.14 (0.02)**	**-0.03 (0.01)**	**0.00 (0.01)**
**Experiment 4**	**0.52 (N/A)**	**0.26 (0.04)**	**0.00 (0.01)**	**0.00 (0.01)**
**Experiment 5**	**N/A**	**0.18 (0.02)**	**-0.11 (0.01)**	**0.00 (0.02)**
**Experiment 6**	**0.21 (0.05)**	**0.08 (0.02)**	**0.00 (0.02)**	**0.00 (0.01)**

## Discussion

We seeded DRG on one side of an MEA and cultured the tissue for a period of 2 days. Using CLEAN electrodes as a reference for resistance values, we found that after two days any electrode with a change of at least 0.15 MΩ more than the average resistance value the CLEAN electrodes would predict *with extremely high accuracy (nearly 100%)* DRG neurite contact with an electrode (Fig 3B). This criterion of 0.15 MΩ would be time dependent and only an accurate predictor of DRG neurite contact with an electrode at the two day time point. If culturing DRG tissue for longer periods of time (or shorter), the criterion would need to be recalculated as resistance values on CLEAN electrodes cultured for more than two days may be different. There are a number of variables that might contribute to the net change in resistance on CLEAN electrodes during the culture process. Specifically, the adsorbed collagen coating on the electrodes required for DRG seeding presumably is changing over time. The rate of this change is currently unknown and may be influenced by a number of factors including the volume of medium in the chamber, the Vroman effect of displacement by other proteins in the media [14], medium agitation from moving the MEA and/or from medium exchanges (both required for long-term culturing of DRG tissue).

In this report, we demonstrate non-invasive and non-destructive evaluation of nerve growth using direct resistance measurements of MEA. The use of resistance spectroscopy has been previously investigated on electrode arrays, with clear ability to detect serum deposition [15, 16], cell culture changes [15, 17], and neural cell growth [16] by the increases in resistance of the electrodes. Our results agree with these reports, with increases in resistance over time in culture and when contacted by neural tissue. However, this technique provided a simplified measurement of resistance, allowing for use across labs without extensive equipment or computational analysis. The experiments outlined in this study were conducted by manually measuring resistance values on 59 extracellular electrodes with a multimeter at discrete time points. By developing an automated system to continuously measure resistance values on all electrodes and cross-referencing those with electrodes known to not be in contact with a DRG neurite on the opposite side of the MEA (CLEAN electrodes), criterion resistance values would be able to reliably detect DRG tissue contact in real-time. This technique would function in lieu of visually identifying DRG tissue in contact with electrodes and pave the way for the development of an autonomous feedback system to continuously monitor DRG neurite outgrowth.

## Abbreviations

DRG: dorsal root ganglia
MEA: microelectrode array
